# Commentary: Lactate-Induced Glucose Output Is Unchanged by Metformin at a Therapeutic Concentration—A Mass Spectrometry Imaging Study of the Perfused Rat Liver

**DOI:** 10.3389/fphar.2019.00090

**Published:** 2019-02-19

**Authors:** Hartmut H. Glossmann, Oliver M. D. Lutz

**Affiliations:** ^1^Institute for Biochemical Pharmacology, Medical University of Innsbruck, Innsbruck, Austria; ^2^Austrian Drug Screening Institute GmbH, Innsbruck, Austria

**Keywords:** metformin, mitochondrial glycerophosphate dehydrogenase, MGPD, fasting plasma glucose (FPG), gluconeogenesis, diabetes

Calza et al. (2018) challenge a widely accepted view (Petersen et al., 2017) that mitochondrial glycerophosphate dehydrogenase (mGPD, EC 1.1.5.3), a component of the glycerolphosphate shuttle (GPS), is the primary molecular target (Madiraju et al., 2014) for the antidiabetic drug metformin to lower plasma glucose in type 2 diabetic patients. The initial report was followed by a detailed preclinical investigation in which the hypothesis that the cellular redox state (i.e., the cellular [NADH]/[NAD^+^] ratio) increased by metformin's selective mGPD inhibition, explained decreased liver gluconeogenesis from lactate and glycerol whereas pyruvate or alanine as precursors were not affected (Madiraju et al., 2018). Already in 1969, Krebs et al. (1969) concluded from their experiments with the ethanol-perfused rat liver that the decrease of the [free NAD^+^]/[free NADH] ratio was responsible for the inhibition of gluconeogenesis from lactate and glycerol whereas pyruvate-driven formation of glucose was completely unaffected. Thus, the evidence for a precursor-selective inhibition of gluconeogenesis by the cellular redox ratio was presented 50 years ago and confirmed for metformin (Madiraju et al., 2014, 2018). The crucial main point is that the claimed primary molecular target for the drug responsible for the ratio change is mGPD. Alternative opinions (e.g., Hunter et al., 2018) to explain reduced liver gluconeogenesis by metformin will not be discussed herein.

The main observations by Madiraju et al. (2014) were that metformin i.v. injections into fasting healthy rats with doses of 20 and 50 mg/kg led, within 30 min, to lowering of fasting plasma glucose (FPG) and endogenous glucose production (EGP), as measured by labeled glucose. Increased ratios in plasma and liver for [lactate]/[pyruvate] as well as decreased ratios of [β-hydroxybutyrate]/[acetoacetate] were seen. Similar observations were made upon chronic i.p. metformin dosing. Thus, acute or chronic metformin inhibits EGP, which is accompanied by changes in plasma and liver with increased [lactate]/[pyruvate] ratios as a proxy for the cytosolic [NADH]/[NAD^+^] redox state. These results in the preclinical models are contrary to the evidence from numerous studies in healthy non-diabetic humans, namely that neither acute nor chronic metformin lowers FPG (Sulaiman and Johnson, 1977; Bonora et al., 1984; Nestler et al., 1994; Sambol et al., 1996; Chung et al., 2018; Gormsen et al., 2018) and even increases EGP (Christensen et al., 2015; Konopka et al., 2016).

In order to explain the changed redox ratios the authors investigated the two main shuttles which can transfer cytosolic NADH to mitochondria, namely the malate-aspartate shuttle (MAS) and the GPS. They came to the conclusion that one component of the latter, mGPD, is responsible and a direct molecular target for metformin.

In the context of metformin's actions on mitochondria it is important to note that MAS, in contrast to GPS, can run in the reverse mode (Vancura et al., 2018) if NADH oxidation is impaired by inhibition of the electron transport chain and cytosolic redox ratios are thereby increased. GPS is a much simpler and faster system and consists of two enzymes: the cytosolic glycerol 3-phosphate dehydrogenase (cGPD, EC 1.1.1.8) with NAD^+^/NADH as co-factor [a K_*m*_ value around 30 μM for glycerophosphate (GP)] and mGPD with FAD as co-factor. The mGPD has a high K_*m*_ for GP (2 to 8 mM), is an important reactive oxygen species (ROS) producer (Orr et al., 2012), is stimulated by calcium and catalyzes the “one-way street” reaction from GP to dihydroxyacetone phosphate. Madiraju et al. (2014) reported liver mitochondrial respiration in the presence and absence of 50 μM metformin by addition of pyruvate, malate, and glutamate, followed by rotenone. GP was then added and a 40% inhibition of the GP-stimulated oxygen consumption rate (OCR) was noticed. In figure 2 of their extended data dose responses of OCR with metformin present (between 0.078 and 20 mM) on complex I OCR are shown. There was no inhibition up to 1.25 mM. After addition of rotenone, succinate was added and a significant stimulation (about 40%) for all metformin concentrations was reported. As a crucial step of evidence, the authors investigated the activity of metformin on the kinetics of different preparations of mGPD, including an immunopurified recombinant human mGPD, rat mitochondrial lysates, immunoprecipitated purified rat mGPD and the distantly related purified *Pediococcus* sp. α-glycerophosphate oxidase. In all instances, a non-competitive inhibition was observed with K_*i*_ values of about 50 μM. The authors also performed *in silico* molecular docking simulations with a modified version of the crystal structure of α-glycerophosphate oxidase from *Streptococcus* sp. and suggested that metformin interacted with the FAD binding site.

To summarize: First, the observed acute inhibition of FPG in healthy rats by metformin in the preclinical model was puzzling as there was no parallel in any of the human studies. Second, we were wondering about the significant (40%) stimulation of succinate-driven OCR by metformin that has never been reported before. Third, high throughput screening for mGPD inhibitors with several thousands of chemicals yielded membrane permeable, high-affinity blockers such as iGP (STK017597, Vitas-M) but not metformin or related structures (Orr et al., 2014). Fourth, mGPD was not among the 41 computationally predicted biomolecular metformin targets (Cuyas et al., 2018). Consequently, we were searching for confirmations of the most important argument for the hypothesis that mGPD is a primary, direct molecular target of metformin but to our dismay, we did not find any. Instead, we discovered convincing proof that metformin does not inhibit mGPD. The first report by one of the laboratories with decades of experience with mGPD did not find any metformin inhibition up to 10 mM but, as a positive control, micromolar inhibition by iGP (Pecinova et al., 2017). The second report is a detailed study on the effects of metformin on the OCR of saponin-permeabilized ACR 549 cancer cells exposed to metformin. With complex I substrates, addition of metformin (1 mM) completely blocked OCR. With GP as substrate, metformin did not inhibit at all (Gui et al., 2016). The third article (Al-Oanzi et al., 2017) investigated concentrations of the immediate intracellular precursor of free glucose in plasma, namely glucose-6-phosphate (G6P) and fructose-2,6-bisphosphate (F26P) in isolated rat hepatocytes. When comparing metformin to iGP, the authors found that in the presence of an inhibitor of G6Pase, metformin (0.1 to 0.5 mM) dose-dependently decreased G6P but iGP had no effect even at 40 μM. Metformin also reduced levels of F26P as proxy for fructose-6-phosphate in agreement with the report by Hunter et al. (2018).

Thus, the data by Calza et al. (2018) and the evidence presented above seriously challenge the “primary, simple, and elegant” (Ferrannini, 2014) metformin target.

## Author's Note

After submission of the manuscript two publications came to our attention, which support the notion that mGPD must be removed from the list of direct molecular metformin targets. Alshawi and Agius (2018) report that metformin (0.1-5 mM) does not inhibit mGPD in permeabilized rodent hepatocytes. Li et al. (2018) discovered that cytochrome C reduction, used as the activity reporter for mGPD in one of the assays by Madiraju et al. (2014), is inhibited by metformin in a concentration-dependent (0.2-2.5 mM) manner.

## Author Contributions

HG and OL discussed the topic and wrote the paper.

### Conflict of Interest Statement

The authors declare that the research was conducted in the absence of any commercial or financial relationships that could be construed as a potential conflict of interest.

## References

[B1] Al-Oanzi Z. H. Fountana S. Moonira T. Tudhope S. J. Petrie J. L. Alshawi A.. (2017). Opposite effects of a glucokinase activator and metformin on glucose-regulated gene expression in hepatocytes. Diabetes Obes. Metab. 19, 1078–1087. 10.1111/dom.12910 28206714

[B2] Alshawi A. Agius L. (2018). Low metformin causes a more oxidized mitochondrial NADH/NAD redox state in hepatocytes and inhibits gluconeogenesis by a redox-independent mechanism. J Biol Chem. 10.1074/jbc.RA118.006670 30591586 PMC6393620

[B3] Bonora E. Cigolini M. Bosello O. Zancanaro C. Capretti L. Zavaroni I.. (1984). Lack of effect of intravenous metformin on plasma concentrations of glucose, insulin, C-peptide, glucagon and growth hormone in non-diabetic subjects. Curr. Med. Res. Opin. 9, 47–51. 10.1185/03007998409109558 6373159

[B4] Calza G. Nyberg E. Makinen M. Soliymani R. Cascone A. Lindholm D.. (2018). Lactate-induced glucose output is unchanged by metformin at a therapeutic concentration - A mass spectrometry imaging study of the perfused rat liver. Front. Pharmacol. 9:141. 10.3389/fphar.2018.00141 29520235 PMC5827415

[B5] Christensen M. M. Hojlund K. Hother-Nielsen O. Stage T. B. Damkier P. Beck-Nielsen H.. (2015). Endogenous glucose production increases in response to metformin treatment in the glycogen-depleted state in humans: a randomised trial. Diabetologia 58, 2494–2502. 10.1007/s00125-015-3733-2 26271344

[B6] Chung H. Oh J. Yoon S. H. Yu K. S. Cho J. Y. Chung J. Y. (2018). A non-linear pharmacokinetic-pharmacodynamic relationship of metformin in healthy volunteers: an open-label, parallel group, randomized clinical study. PLoS ONE 13:e0191258. 10.1371/journal.pone.0191258 29342199 PMC5771593

[B7] Cuyas E. Verdura S. Llorach-Pares L. Fernandez-Arroyo S. Luciano-Mateo F. Cabre N.. (2018). Metformin directly targets the H3K27me3 demethylase KDM6A/UTX. Aging Cell 17:e12772. 10.1111/acel.12772 29740925 PMC6052472

[B8] Ferrannini E. (2014). The target of metformin in type 2 diabetes. N. Engl. J. Med. 371, 1547–1548. 10.1056/NEJMcibr1409796 25317875

[B9] Gormsen L. C. Sondergaard E. Christensen N. L. Jakobsen S. Nielsen E. H. T. (2018). Metformin does not affect postabsorptive hepatic free fatty acid uptake, oxidation or resecretion in humans: a 3-month placebo-controlled clinical trial in patients with type 2 diabetes and healthy controls. Diabetes Obes. Metab. 20, 1435–1444. 10.1111/dom.13244 29405635

[B10] Gui D. Y. Sullivan L. B. Luengo A. Hosios A. M. Bush L. N. Gitego N.. (2016). Environment dictates dependence on mitochondrial complex I for NAD+ and aspartate production and determines cancer cell sensitivity to metformin. Cell Metab. 24, 716–727. 10.1016/j.cmet.2016.09.006 27746050 PMC5102768

[B11] Hunter R. W. Hughey C. C. Lantier L. Sundelin E. I. Peggie M. Zeqiraj E.. (2018). Metformin reduces liver glucose production by inhibition of fructose-1-6-bisphosphatase. Nat. Med. 24, 1395–1406. 10.1038/s41591-018-0159-7 30150719 PMC6207338

[B12] Konopka A. R. Esponda R. R. Robinson M. M. Johnson M. L. Carter R. E. Schiavon M.. (2016). Hyperglucagonemia mitigates the effect of metformin on glucose production in prediabetes. Cell Rep. 15, 1394–1400. 10.1016/j.celrep.2016.04.024 27160898 PMC4871720

[B13] Krebs H. A. Freedland R. A. Hems R. Stubbs M. (1969). Inhibition of hepatic gluconeogenesis by ethanol. Biochem. J. 112, 117–124. 10.1042/bj1120117 5774487 PMC1187647

[B14] Li X. Wang X. Snyder M. P. (2018). Metformin affects heme function as a possible mechanism of action. G3 10.1534/g3.118.200803 30554148 PMC6385989

[B15] Madiraju A. K. Erion D. M. Rahimi Y. Zhang X. M. Braddock D. T. Albright R. A.. (2014). Metformin suppresses gluconeogenesis by inhibiting mitochondrial glycerophosphate dehydrogenase. Nature 510, 542–546. 10.1038/nature13270 24847880 PMC4074244

[B16] Madiraju A. K. Qiu Y. Perry R. J. Rahimi Y. Zhang X. M. Zhang D.. (2018). Metformin inhibits gluconeogenesis via a redox-dependent mechanism *in vivo*. Nat. Med. 24, 1384–1394. 10.1038/s41591-018-0125-4 30038219 PMC6129196

[B17] Nestler J. E. Beer N. A. Jakubowicz D. J. Beer R. M. (1994). Effects of a reduction in circulating insulin by metformin on serum dehydroepiandrosterone sulfate in nondiabetic men. J. Clin. Endocrinol. Metab. 78, 549–554. 8126125 10.1210/jcem.78.3.8126125

[B18] Orr A. L. Ashok D. Sarantos M. R. Ng R. Shi T. Gerencser A. A.. (2014). Novel inhibitors of mitochondrial sn-glycerol 3-phosphate dehydrogenase. PLoS ONE 9:e89938. 10.1371/journal.pone.0089938 24587137 PMC3933693

[B19] Orr A. L. Quinlan C. L. Perevoshchikova I. V. Brand M. D. (2012). A refined analysis of superoxide production by mitochondrial sn-glycerol 3-phosphate dehydrogenase. J. Biol. Chem. 287, 42921–42935. 10.1074/jbc.M112.397828 23124204 PMC3522288

[B20] Pecinova A. Drahota Z. Kovalcikova J. Kovarova N. Pecina P. Alan L.. (2017). Pleiotropic effects of biguanides on mitochondrial reactive oxygen species production. Oxid. Med. Cell Longev. 2017:7038603. 10.1155/2017/7038603 28874953 PMC5569935

[B21] Petersen M. C. Vatner D. F. Shulman G. I. (2017). Regulation of hepatic glucose metabolism in health and disease. Nat. Rev. Endocrinol. 13, 572–587. 10.1038/nrendo.2017.80 28731034 PMC5777172

[B22] Sambol N. C. Chiang J. O'Conner M. Liu C. Y. Lin E. T. Goodman A. M.. (1996). Pharmacokinetics and pharmacodynamics of metformin in healthy subjects and patients with noninsulin-dependent diabetes mellitus. J. Clin. Pharmacol. 36, 1012–1021. 10.1177/009127009603601105 8973990

[B23] Sulaiman W. R. Johnson R. H. (1977). The effects of phenformin and metformin on fat and carbohydrate metabolism. Acta Diabetol. Lat. 14, 129–136. 10.1007/BF02581400 605743

[B24] Vancura A. Bu P. Bhagwat M. Zeng J. Vancurova I. (2018). Metformin as an anticancer agent. Trends Pharmacol. Sci. 39, 867–878. 10.1016/j.tips.2018.07.006 30150001 PMC6153060

